# Genetic parameters for automatically-measured vaginal temperature, respiration efficiency, and other thermotolerance indicators measured on lactating sows under heat stress conditions

**DOI:** 10.1186/s12711-023-00842-x

**Published:** 2023-09-20

**Authors:** Pedro H. F. Freitas, Jay S. Johnson, Hui Wen, Jacob M. Maskal, Francesco Tiezzi, Christian Maltecca, Yijian Huang, Ashley E. DeDecker, Allan P. Schinckel, Luiz F. Brito

**Affiliations:** 1 Department of Animal Sciences, Purdue University, West Lafayette, IN USA; 2 USDA-ARS Livestock Behavior Research Unit, West Lafayette, IN USA; 3 Department of Animal Science, North Carolina State University, Raleigh, NC USA; 4 Department of Agriculture, Food, Environment and Forestry, University of Florence, Florence, Italy; 5 Smithfield Premium Genetics, Rose Hill, NC USA; 6 Smithfield Foods, Inc., Warsaw, NC USA

## Abstract

**Background:**

Genetic selection based on direct indicators of heat stress could capture additional mechanisms that are involved in heat stress response and enable more accurate selection for more heat-tolerant individuals. Therefore, the main objectives of this study were to estimate genetic parameters for various heat stress indicators in a commercial population of Landrace × Large White lactating sows measured under heat stress conditions. The main indicators evaluated were: skin surface temperatures (SST), automatically-recorded vaginal temperature (T_V_), respiration rate (RR), panting score (PS), body condition score (BCS), hair density (HD), body size (BS), ear size, and respiration efficiency (R_eff_).

**Results:**

Traits based on T_V_ presented moderate heritability estimates, ranging from 0.15 ± 0.02 to 0.29 ± 0.05. Low heritability estimates were found for SST traits (from 0.04 ± 0.01 to 0.06 ± 0.01), RR (0.06 ± 0.01), PS (0.05 0.01), and R_eff_ (0.03 ± 0.01). Moderate to high heritability values were estimated for BCS (0.29 ± 0.04 for caliper measurements and 0.25 ± 0.04 for visual assessments), HD (0.25 ± 0.05), BS (0.33 ± 0.05), ear area (EA; 0.40 ± 0.09), and ear length (EL; 0.32 ± 0.07). High genetic correlations were estimated among SST traits (> 0.78) and among T_V_ traits (> 0.75). Similarly, high genetic correlations were also estimated for RR with PS (0.87 ± 0.02), with BCS measures (0.92 ± 0.04), and with ear measures (0.95 ± 0.03). Low to moderate positive genetic correlations were estimated between SST and T_V_ (from 0.25 ± 0.04 to 0.76 ± 0.07). Low genetic correlations were estimated between T_V_ and BCS (from − 0.01 ± 0.08 to 0.06 ± 0.07). Respiration efficiency was estimated to be positively and moderately correlated with RR (0.36 ± 0.04), PS (0.56 ± 0.03), and BCS (0.56 ± 0.05 for caliper measurements and 0.50 ± 0.05 for the visual assessments). All other trait combinations were lowly genetically correlated.

**Conclusions:**

A comprehensive landscape of heritabilities and genetic correlations for various thermotolerance indicators in lactating sows were estimated. All traits evaluated are under genetic control and heritable, with different magnitudes, indicating that genetic progress is possible for all of them. The genetic correlation estimates provide evidence for the complex relationships between these traits and confirm the importance of a sub-index of thermotolerance traits to improve heat tolerance in pigs.

**Supplementary Information:**

The online version contains supplementary material available at 10.1186/s12711-023-00842-x.

## Background

Heat stress is a major welfare and production concern in the swine industry that negatively impacts metabolism and physiological mechanisms [1] of the animals themselves (direct effect [2, 3]) or of their offspring due to in-utero heat stress [4]. When experiencing adverse climatic conditions, animals can exhibit a range of biological responses to counteract the excess of body heat, including reducing feed intake, altering feeding behavior, increasing respiration rate, increasing blood flow to the skin, and other behavioral changes [3, 5, 6]. In a recent study, Johnson et al. [7] reported that sows begin to suffer from moderate heat stress at temperatures above 26.6 °C and may be severely affected at temperatures above 29.4 °C.

Advances in genetic and genomic selection, nutrition practices, and management have increased sow performance, including larger litter sizes and higher milk production [8]. These changes have, in turn, resulted in higher metabolic heat production [9, 10]. To date, heat tolerance has rarely been included in swine selection indexes in worldwide breeding programs. In addition, to date, most genetic analyses for thermotolerance in pigs have been based on routinely recorded traits (e.g., body weight, carcass weight, reproduction) and data from public weather stations [11–15]. This approach can be incorporated in existing breeding programs and capture genetic variability in thermotolerance between individuals [16]. However, as pigs are mostly raised in barns that typically adopt heat mitigation strategies, the climatic variables recorded by public weather stations may not properly reflect the conditions experienced by the animals within barns. Furthermore, the indicators of production efficiency used to calculate individual thermotolerance do not capture the substantial variation in the biological mechanisms involved in heat stress response that is less dependent on production variables [16]. Although efforts have been made to identify novel phenotypes under non-commercial conditions based on simulated heat stress conditions [17, 18], these studies have not been implemented in commercial conditions.

Our hypothesis is that genetic selection based on closer-to-biology indicators of heat stress can capture additional physiological and behavioral mechanisms involved in heat stress response and enable more accurate selection of individuals adapted to challenging climatic conditions. In spite of its importance, physiological indicators of heat stress are not regularly collected in swine herds due to measurement difficulties, need of specific personnel training, and costs. For example, core body temperature would be an ideal metric, however it is difficult and usually invasive to measure. To overcome these difficulties, several approaches to measure body temperature have been developed in recent years, such as intramuscular implants in broilers [19, 20], rumen boluses in cattle [21, 22], and wearable sensors for recording vaginal temperature in cattle [23, 24] and pigs [25]. Notably, most studies that have evaluated body temperature in livestock species have been based on a small number of observations per individual [26]. However, body temperature (rectal, vaginal) changes throughout the day and therefore, a large environmental effect is usually observed (lowering heritability estimates). In this context, automatically-measured body temperature could better capture its variability during heat stress conditions.

Genetic selection for more heat-tolerant animals has the potential to improve animal welfare, productive efficiency, and the long-term sustainability of the swine industry, especially considering that the effects of climate change are becoming more severe [27, 28]. Therefore, the main objectives of this study were to identify statistical models and estimate genetic parameters (including heritabilities and genetic correlations) for various heat stress indicators in a commercial population of lactating Landrace x Large White sows measured under heat stress conditions. The main indicators evaluated were skin surface temperatures (SST), vaginal temperature (T_V_), respiration rate (RR), panting score (PS), body condition scores (BCS), hair density (HD), body size (BS), ear measurements, and respiration efficiency (R_eff_).

## Methods

The experimental protocol followed ethical principles in animal research (Federation of Animal Science Societies, 2020) and was approved by the Purdue University Animal Care and Use Committee (Protocol #1912001990).

### Animals and genotypes

Phenotypes were collected on 1645 multiparous (from parity 2 to 7) lactating sows (Large White x Landrace cross) under commercial settings (lower use of heat abatement strategies), as described by Freitas [29] and Johnson et al. [7]. In total, 1639 animals were genotyped using the PorcineSNP50K [50,703 single nucleotide polymorphisms (SNPs)] Bead Chip (Illumina, San Diego, CA, USA). Quality control (QC) of the genotype data consisted of removing SNPs and animals with a call rate lower than 0.90, a minor allele frequency lower than 0.01, and a difference between observed and expected heterozygous frequencies higher than 0.15. Genomic QC was applied using the BLUPF90 family software [30]. After genomic QC, 49,547 SNPs for 1625 animals were kept for subsequent analyses.

### Environmental data

For in-barn environmental data, 4 data loggers (Hobo model #MX1101; data logger temperature/Relative humidity; accuracy ± 0.20 °C and ± 2% RH; Onset; Bourne, MA) per farrowing room were placed in one of 4 quadrants that were mounted at sow height, and dry bulb temperature (DBT) and relative humidity (RH) were recorded every five minutes during the data collection period (June 9, 2021 and July 24, 2021). Using the in-barn climatic variable record, dew point (DP) was calculated using the Magnus-Tetens equation [31] as follows:

1 $$\mathrm{DP}=(\mathrm{b\alpha }({\mathrm{DBT}}_{\mathrm{a}},\mathrm{RH}))/(\mathrm{a}-\mathrm{\alpha }\left({\mathrm{DBT}}_{\mathrm{a}},\mathrm{RH}\right),$$

where $\mathrm{a}=17.62,\mathrm{b}=243.12\mathrm{^\circ{\rm C} }$, and $\mathrm{\alpha }\left({\mathrm{DBT}}_{\mathrm{a}},\mathrm{RH}\right)=\mathrm{ln}\left(\frac{\mathrm{RH}}{100}\right)+\frac{{\mathrm{aDBT}}_{\mathrm{a}}}{\mathrm{b}+{\mathrm{DBT}}_{\mathrm{a}}}$. The 5 min interval records for DBT, RH, and DP were used to calculate the average hourly temperature (AVGtemp), average hourly RH (AVG_RH_), and average hourly DP (AVG_DP_) for statistical analyses by averaging all recorded points during one hour. The environmental data from each of the 4 data loggers (or in some cases each of the remaining ones in the room when some of them stopped working during the experiment) were used to calculate the averaged climatic variables.

### Thermoregulatory indicators

Thermoregulatory indicators of heat stress were collected from June 9 to July 24, 2021, and are described in [7]. In summary, the phenotypes consisted of respiration rate, PS, ear skin temperature (T_ES_), shoulder skin temperature (T_SS_), rump skin temperature (T_RS_), tail skin temperature (T_TS_), and T_V_, which were collected on all sows throughout the study. RR was collected by counting flank movements for 15 s at 8:00, 12:00, 16:00, and 20:00 h daily during 4 consecutive days, and multiplied by 4 to calculate breaths per min (bpm) [32, 33]. Panting score was collected daily during 4 consecutive days at 15:30 h and was scored from 0 to 3 (i.e., score 0 for animals with a closed mouth and normal breathing; 1 for animals with a closed mouth and rapid breathing; 2: for animals with an open mouth and rapid breathing; and 3: for animals with an open mouth and rapid breathing with obvious salivation). SST was measured using an infrared temperature gun at 8:00, 12:00, 16:00, and 20:00 h daily during 4 consecutive days.

**Fig. 1:**
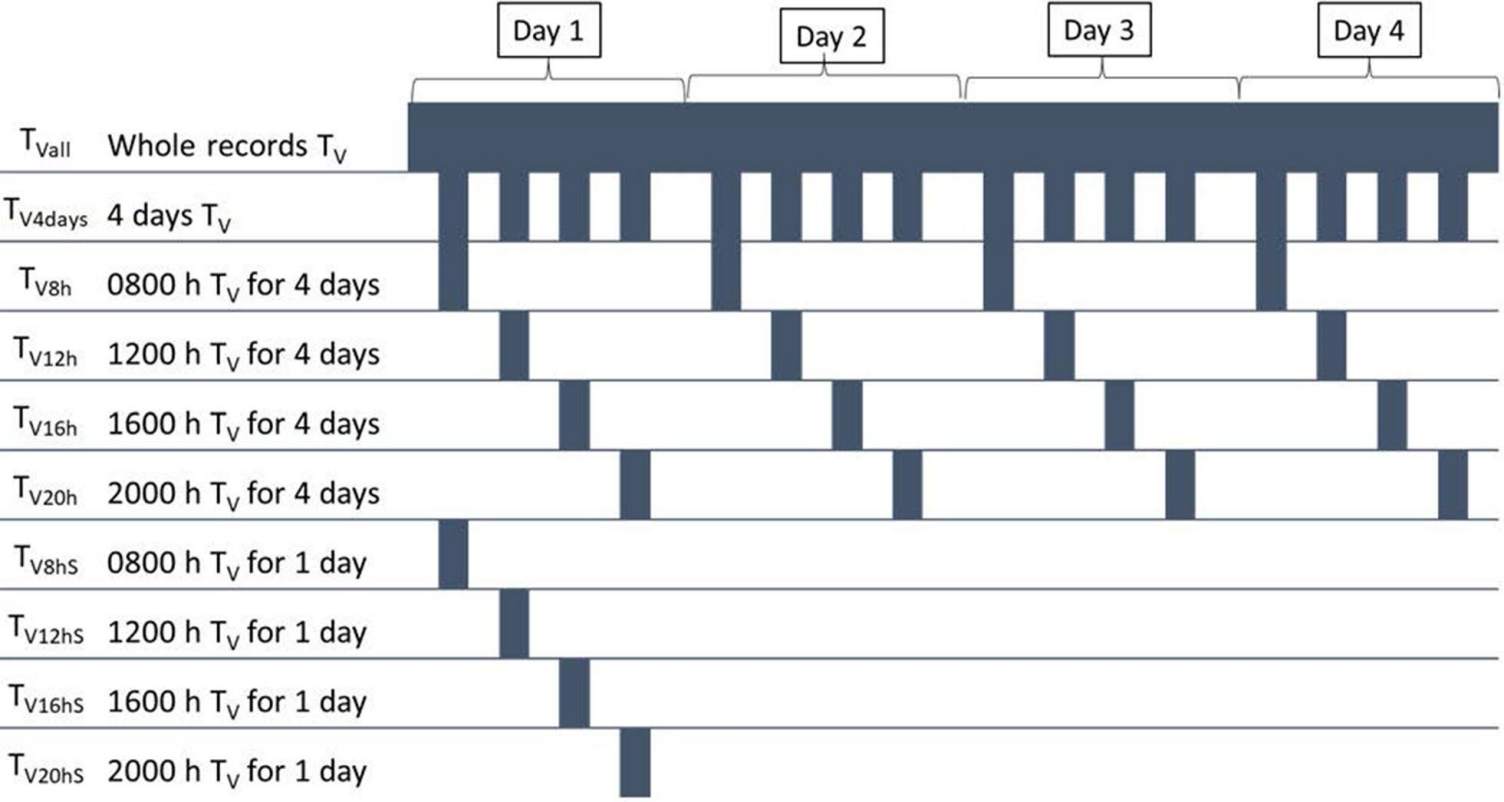
Scheme representing the subsets for vaginal temperature measures. Graphic representation of all datasets derived from the automatically-measured vaginal temperature. T_Vall_: corresponds to whole data measured every 10 min; T_V4days_: 4 measurements at 8:00, 12:00, 16:00, and 20:00 h during the 4 days; T_V8h_. T_V12h_, T_V16h_, T_V20h_: hourly daily measure for the 4 collection days at 8:00, 12:00, 16:00, and 20:00 h, respectively; T_V8hS_. T_V12hS_, T_V16hS_, T_V20hS_: single record day measured on the first day of collection at 8:00, 12:00, 16:00, and 20:00 h, respectively

Vaginal temperature was monitored in 10-min intervals using the calibrated thermochron temperature, as described in previous reports [23, 25]. The vaginal monitors were removed at the end of the data collection period. Different sets of traits were developed from the T_V_ measured every 10 min, as shown in Fig. 1. In summary, the traits derived from the T_V_ were: data measured every 10 min (T_Vall_) and traits derived from T_Vall_ by averaging the 6 records per hour. These new derived traits included vaginal temperature based on: four-time measurements corresponding to records at 8:00, 12:00, 16:00, and 20:00 h during the 4 days (T_V4days_), hourly daily measures for the 4 collection days corresponding to records at 8:00, 12:00, 16:00, and 20:00 h (T_V8h_, T_V12h_, T_V16h_, and T_V20h_, respectively), and single-records corresponding to measurements taken at 8:00, 12:00, 16:00, and 20:00 h on the first day of collection (T_V8hS_. T_V12hS_, T_V16hS_, and T_V20hS_, respectively). In addition, R_eff_ was derived as the regression slope of T_V_ on RR, using records of RR measured at 8:00 and 16:00 h and the corresponding hourly T_V_ (average of six records per hour). The use of these two time-points corresponded to the beginning of the measurement day (i.e., at 8:00 h) and the hottest period of the day (here represented by the measurement at 16:00 h).

For each body temperature phenotype (i.e., T_ES_, T_SS_, T_RS_, T_TS_, and T_V_), outliers were removed if they deviated by more than 3.5 standard deviation (SD) from the trait mean. For RR, data below 12 bpm and above 172 bpm were also removed. The total number of records and animals for each trait is in Table 1.

**Table 1:** Total number of records, number of animals, and effects for each trait measured in lactating sows under heat stress conditions

Trait	Number of records	Number of animals with records	Systematic effects	Random effects
T_ES_	25,568	1645	TREC, WDT, PAR, DIL, LOC, CLIM	a, pe
T_SS_	25,572	1645	TREC, WDT, PAR, DIL, LOC, CLIM	a, pe
T_RS_	25,571	1643	TREC, WDT, PAR, DIL, LOC, CLIM	a, pe
T_TS_	25,570	1643	TREC, WDT, PAR, DIL, LOC, CLIM	a, pe
T_Vall_	932,926	1381	WD, PAR, LOC, CLIM	a, pe
T_V4days_	21,415	1381	WDT, PAR, DIL, LOC, CLIM	a, pe
T_V8h_	5358	1381	WD, PAR, DIL, LOC, CLIM	a, pe
T_V12h_	5349	1381	WD, PAR, DIL, LOC, CLIM	a, pe
T_V16h_	5359	1381	WD, PAR, DIL, LOC, CLIM	a, pe
T_V20h_	5349	1381	WD, PAR, DIL, LOC, CLIM	a, pe
T_V8hS_	1381	1381	W, PAR, DIL, LOC, CLIM	a
T_V12hS_	1381	1381	W, PAR, DIL, LOC, CLIM	a
T_V16hS_	1381	1381	W, PAR, DIL, LOC, CLIM	a
T_V20hS_	1381	1381	W, PAR, DIL, LOC, CLIM	a
RR	25,815	1643	TREC, WDT, PAR, DIL, LOC, CLIM	a, pe
PS	6577	1642	TREC, WD, PAR, DIL, LOC, CLIM	a, pe
R_eff_	1381	1381	PAR	a
BCS_Cal_	1615	1615	TREC, W, PAR, LOC, DIL	a
BCS_Vis_	1598	1598	TREC, W, PAR, LOC, DIL	a
HD	1344	1344	TREC, PAR	a
BS	1639	1639	TREC, W, PAR	a
EA	705	705	TREC, PQ	a
EL	713	713	TREC, PQ	a

*T*_*ES*_ ear skin temperature (°C), *T*_*SS*_ shoulder skin temperature (°C), *T*_*RS*_ rump skin temperature (°C), *T*_*TS*_ tail skin temperature (°C), *T*_*V4all*_ all measures (every 10 min) of vaginal temperatures during 4 days (°C), *T*_*V4days*_ four time-point measures of vaginal temperatures during 4 days (°C), *T*_*V8h*_ vaginal temperature measured at 8:00 h, during 4 days (°C), T_V12h_: vaginal temperature measured at 12:00 h, during 4 days (°C), *T*_*V16h*_ vaginal temperature measured at 16:00 h, during 4 days (°C), *T*_*V20h*_ vaginal temperature measured at 20:00 h, during 4 days, *RR* (°C): respiration rate/minute, *PS* panting score; *R*_*eff*_ respiration efficiency, *BCS*_*Cal*_ caliper body condition score, *BCS*_*Vis*_ visual body condition score, *HD* hair density score, *BS* body size score, *EA* ear area (cm^2^), *EL* ear length (cm)

*TREC* trait recorder, *WDT* concatenation of week, day, and time of measurement, *WD* concatenation of week and day of measurement, *W* week of measurement, *PAR* parity, *DIL* days in lactation, *LOC* concatenation of barn type and room, *CLIM* in-barn environmental variable, *PQ* picture quality (e.g., hole, missing piece) *a* random animal genetic effect, *pe* random permanent environmental effect

### Anatomical characteristics

Anatomical characteristics associated with heat dissipation capacity were recorded on all sows, including ear size, HD, BCS, and BS. For measures of ear size, a 10.2 × 15.2 cm grid card containing 1 × 1 cm squares was placed next to the sows’ ear and a photo was taken with a digital camera for later analyses of ear area (cm^2^; EA) and ear length (cm; EL) using Image J (National Institutes of Health; Bethesda, MD, USA). Pictures of damaged ears (e.g., missing pieces of tissue) were excluded from the analyses. Hair density was evaluated using a visual score from 0 to 2 (with 0 for hairless sows or sows with a small amount of hair; 1 for sows with normal (moderate) hair cover; and, 2 for sows with substantial hair cover and long hair length). Body condition was scored using a caliper tool (BCS_Cal_; [34]) and visually (BCS_Vis_) using a scale from 1 to 3, with score 1 for a thin animal with landmark bones that were prominent with or without a slight hand pressure; score 2 for an ideal animal, with bones that were barely felt when palpating with firm pressure; and score 3 for an animal without visible bones and that were undetectable by palpation. The original BCS_Cal_ scale (i.e., thin, ideal, and fat) was transformed to a continuous scale from 1 to 15, with 1 to 8, 9 to 12, and 13 to 15 corresponding to the original “thin”, “ideal”, and “fat” categories, respectively. Individual sows were also visually scored by two independent researchers into three categories (i.e., small, medium, or large) according to their BS. The total number of records and animals for each trait is in Table 1.

### Statistical analyses

The systematic effects used in the statistical model to describe each trait were defined based on the backward elimination procedure (P < 0.05) using the *lm* function available in the R software [35]. After defining the systematic effects, the impact of fitting each of three climate variables [i.e., RH, average temperature (AVG_temp_), and DP] were subjected to single-trait model comparisons based on the deviance information criterion (DIC) using the THRGIBBS1F90 software [36]. The final model for each trait is given in Table 1. Subsequent bivariate analyses were also performed using the THRGIBBS1F90 software [36]. A chain containing 100,000 iterations, with burn-in and thinning of 40,000 and 30, respectively, were used for all trait combinations and allowed model convergence for all analyses, as assessed based on graphical analyses and the Raftery and Lewis criterion [37] implemented in the Bayesian Output Analysis [38] package of the R software [35]. Traits with repeated records (T_ES_, T_SS_, T_RS_, T_TS_, T_V_, and RR) were analyzed using a repeatability model and single-record traits were analyzed using an animal model. The statistical models for repeated [Eq. (2) and single record traits (Eq. (3)] can be described as:

2 $$\mathbf{y}=\mathbf{X}{\varvec{\upbeta}}+{\mathbf{Z}}_{\mathbf{a}}\mathbf{a}+{\mathbf{Z}}_{\mathbf{p}\mathbf{e}}\mathbf{p}\mathbf{e}+\mathbf{e},$$

3 $$\mathbf{y}=\mathbf{X}{\varvec{\upbeta}}+{\mathbf{Z}}_{\mathbf{a}}\mathbf{a}+\mathbf{e},$$

where $\mathbf{y}$ is the vector of phenotypic records; ${\varvec{\upbeta}}$ is the vector of fixed effects specific to each trait, as listed in Table 1; $\mathbf{a}$ is the vector of random animal genetic effects, assumed to follow $\mathbf{a}\sim N(0,\mathbf{G}{\upsigma }_{\mathrm{a}}^{2})$, where ${\upsigma }_{\mathrm{a}}^{2}$ is the additive genetic variance. The $\mathbf{G}$ matrix was created with genomic information using the first method proposed by [39]; $\mathbf{p}\mathbf{e}$ is the vector of random permanent environmental effects, defined as $\mathbf{p}\mathbf{e}\sim N(0,\mathbf{I}{\upsigma }_{\mathrm{pe}}^{2})$, where ${\upsigma }_{\mathrm{pe}}^{2}$ is the permanent environmental variance; $\mathbf{X}$, ${\mathbf{Z}}_{\mathbf{a}}$, and ${\mathbf{Z}}_{\mathbf{p}\mathbf{e}}$ are incidence matrices; and $\mathbf{e}$ is the vector of residual effects assumed to follow $\mathbf{e}\sim N(0,\mathbf{I}{\upsigma }_{\mathrm{e}}^{2})$, where ${\upsigma }_{\mathrm{e}}^{2}$ is the residual variance. Estimates of the heritability (h2; Eq. (4)) and repeatability [r_e_; Eq. (5)] for each trait were calculated as follows:

4 $${\mathrm{h}}^{2}=\frac{{\widehat{\sigma }}_{a}^{2}}{{\widehat{\sigma }}_{a}^{2}+{\widehat{\sigma }}_{pe}^{2}+ {\widehat{\sigma }}_{e}^{2}},$$

5 $${\mathrm{r}}_{\mathrm{e}}=\frac{{\widehat{\sigma }}_{a}^{2}+{\widehat{\sigma }}_{pe}^{2}}{{\widehat{\sigma }}_{a}^{2}+{\widehat{\sigma }}_{pe}^{2}+{\widehat{\sigma }}_{e}^{2}},$$

where ${\widehat{\sigma }}_{a}^{2}$ is the estimate of the additive genetic variance, ${\widehat{\sigma }}_{pe}^{2}$ is the permanent environmental variance (equal to 0 for the single record traits), and ${\widehat{\sigma }}_{e}^{2}$ is the residual variance. Genetic correlations (${\mathrm{r}}_{\mathrm{g}}$) between each pair of traits, following the same type of model as described above, and calculated as follows:

6 $${\mathrm{r}}_{\mathrm{g}}=\frac{{\mathrm{cov}}_{12}}{\sqrt{({\widehat{\sigma }}_{a1}^{2})({\widehat{\sigma }}_{a2}^{2})}},$$

where ${\mathrm{r}}_{\mathrm{g}}$ is the genetic correlation, ${\mathrm{cov}}_{12}$ is the genetic covariance between trait 1 and trait 2, ${\widehat{\sigma }}_{a1}^{2}$ is the additive genetic variance of trait 1, and ${\widehat{\sigma }}_{a2}^{2}$ is the additive genetic variance of trait 2.

The theoretical accuracy of the breeding values predicted for each trait and animal was calculated as:

7 $${\mathrm{Accuracy}}_{\mathrm{i}}=\sqrt{1-\frac{{\widehat{\mathrm{SD}}}_{\mathrm{i}}^{2}}{(1+{\mathrm{F}}_{\mathrm{i}}){\widehat{\upsigma }}_{\mathrm{a}}^{2}}},$$

where $\widehat{{\mathrm{SD}}_{\mathrm{t}}}$ is the posterior standard deviation of the sampled true breeding value (i.e., GEBV) for animal $\mathrm{i}$ estimated based on single-trait analysis, ${\mathrm{F}}_{\mathrm{i}}$ is the genomic inbreeding coefficient based on the diagonal of the genomic relationship matrix, and ${\widehat{\sigma }}_{a}^{2}$ is the estimated additive genetic variance, as above [40].

## Results

### Descriptive statistics of phenotypes and environmental descriptors

Descriptive statistics for the continuous traits (T_ES_, T_SS_, T_RS_, T_TS_, T_V_, RR, EA, and EL) are in Table 2.

**Table 2:** Descriptive statistics for the indicators of heat stress response in lactating sows

Trait	Mean values ± SD	Minimum	Maximum
T_ES_	36.74 ± 1.07	32.50	40.70
T_SS_	36.46 ± 1.08	32.30	39.80
T_RS_	37.23 ± 0.92	33.60	39.90
T_TS_	36.90 ± 0.95	33.20	40.00
T_Vall_	39.74 ± 0.75	37.08	42.72
T_V4days_	39.73 ± 0.77	37.08	42.35
T_V8h_	39.16 ± 0.61	37.23	41.42
T_V12h_	39.57 ± 0.64	37.08	41.78
T_V16h_	40.02 ± 0.69	37.34	42.71
T_V20h_	40.15 ± 0.71	37.61	42.10
T_V8hS_	39.25 ± 0.59	37.42	41.42
T_V12hS_	39.69 ± 0.64	37.08	41.60
T_V16hS_	40.15 ± 0.69	37.34	42.72
T_V20hS_	40.25 ± 0.69	37.84	41.83
RR	73 ± 28	12	172
EA	309.01 ± 53.62	183.23	487.85
EL	24.98 ± 2.81	14.83	34.34

*T*_*ES*_ ear skin temperature (°C), *T*_*SS*_ shoulder skin temperature (°C), *T*_*RS*_ rump skin temperature (°C), *T*_*TS*_ tail skin temperature (°C), *T*_*V4all*_ all measures (each 10 min) of vaginal temperatures during 4 days (°C), T_V4days_: four time measures of vaginal temperatures during 4 days (°C), *T*_*V8h*_ vaginal temperature measured at 8:00 h, during 4 days (°C), *T*_*V12*h_ vaginal temperature measured at 12:00 h, during 4 days (°C); *T*_*V16h*_ vaginal temperature measured at 16:00 h, during 4 days (°C), *T*_*V20h*_ vaginal temperature measured at 20:00 h, during 4 days, *RR* (°C) respiration rate/min, *EA* ear area (cm^2^), *EL* ear length (cm), *SD* standard deviation

### Climate variable covariate selection

Table 3 shows the single trait heritability estimates and the DIC values considering different climate covariates for each trait that had a climate variable included in the model (i.e., T_ES_, T_SS_, T_RS_, T_TS_, T_V_, RR, and PS). AVG_temp_ presented the best estimates of DIC (i.e., the lowest DIC) and the largest amount of estimated additive genetic variance among the available in-barn climate descriptors. Therefore, AVG_temp_ was chosen as the covariate for each trait in the subsequent analyses. A quadratic function of AVG_temp_ was also evaluated as covariates but it presented a worse model fit than the linear covariate of AVG_temp_ for all traits, except for T_SS_ and T_TS_. Therefore, a linear function of AVG_temp_ was included in the model for T_ES_, T_RS_, T_V_, RR, and PS, and a quadratic function of AVG_temp_ for T_SS_ and T_TS_.

**Table 3:** Deviance information criterion (DIC) and estimates of heritability (h^2^) of traits considering average temperature, relative humidity, or dew point as a covariate in the models

Trait	Average temperature		Relative humidity		Dew point	
	h^2^	DIC	h^2^	DIC	h^2^	DIC
T_ES_	0.04 ± 0.01	54,985	0.04 ± 0.01	55,007	0.04 ± 0.01	55,044
T_SS_	0.06 ± 0.01	51,474	0.06 ± 0.01	51,532	0.06 ± 0.01	51,621
T_RS_	0.06 ± 0.01	40,020	0.06 ± 0.01	40,151	0.06 ± 0.01	40,277
T_TS_	0.05 ± 0.01	44,986	0.05 ± 0.01	45,079	0.05 ± 0.01	45,154
T_Vall_	0.15 ± 0.02	1,542,398	0.16 ± 0.03	1542,789	0.17 ± 0.03	1542,566
T_V4days_	0.22 ± 0.03	19,914	0.21 ± 0.03	20,156	0.21 ± 0.03	20,128
RR	0.06 ± 0.01	191,993	0.06 ± 0.01	192,030	0.06 ± 0.01	192,021
PS	0.05 ± 0.01	5313	0.05 ± 0.01	5351	0.05 ± 0.01	5341

*T*_*ES*_ ear skin temperature, *T*_*SS*_ shoulder skin temperature, *T*_*RS*_ rump skin temperature, *T*_*TS*_ tail skin temperature, *T*_*V4all*_ all measures (every10 min) of vaginal temperatures during 4 days, *T*_*V4days*_ four time-point measures of vaginal temperatures during 4 days, *RR* respiration rate/min, *PS* panting score

### Heritability and repeatability

The heritability estimates for each trait are in Table 4. The heritability estimates ranged from 0.03 (R_eff_) to 0.40 (EA). SST traits generally had a low heritability, ranging from 0.04 to 0.06. Low heritability estimates of 0.06 and 0.05 were also observed for RR and PS, respectively. Vaginal temperature based on all records (T_Vall_) had moderate heritability (0.15). In contrast, T_V_ records developed from T_Vall_ had higher heritability estimates. The measures of T_V_ based on repeated records per day for 4 days (i.e., T_V4days_) and the single time per day measures (i.e., T_V8h_, T_V12h_, T_V16h_, and T_V20h_) had moderate heritabilities, with T_V12h_ having the highest heritability (0.24). The estimates of heritability for single record T_V_ traits (T_V8hS_, T_V12hS_, T_V16hS_, and T_V20hS_) were similar to those for the single time per day measures, with T_V12hS_ being the trait with the highest heritability. The single heritability estimates were equal to 0.25, 0.29, 0.22, and 0.22 for T_V8hS_, T_V12hS_, T_V16hS_, and T_V20hS_, respectively. Moderate heritability values of 0.25, 0.29, and 0.25 were observed for BCS_Vis_, BCS_Cal_, and HD, respectively. The body size score was the most heritable trait among those studied, with a heritability of 0.33. Regarding repeatability, T_ES_, T_SS_, T_RS_, T_TS_, RR, and PS had the lowest r_e_ values (0.10, 0.21, 0.22, 0.18, 0.19, and 0.16, respectively), while the traits based on T_V_ had the highest r_e_, ranging from 0.37 (for T_Vall_) to 0.59 (T_V8h_).

**Table 4:** Estimates of additive genetic variance, heritability (± SD), repeatability, and accuracy for each trait measured on lactating sows under heat stress conditions

Trait	Additive genetic variance	Heritability ± SE	Repeatability	Accuracy
T_ES_	0.0313	0.04 ± 0.01	0.10	0.60 ± 0.07
T_SS_	0.0446	0.06 ± 0.01	0.21	0.58 ± 0.07
T_RS_	0.0290	0.06 ± 0.01	0.22	0.59 ± 0.07
T_TS_	0.0298	0.05 ± 0.01	0.18	0.59 ± 0.07
T_Vall_	0.0620	0.15 ± 0.02	0.37	0.66 ± 0.07
T_V4days_	0.0619	0.22 ± 0.03	0.57	0.58 ± 0.06
T_V8h_	0.0633	0.23 ± 0.03	0.59	0.57 ± 0.07
T_V12h_	0.0767	0.24 ± 0.03	0.56	0.60 ± 0.07
T_V16h_	0.0699	0.19 ± 0.02	0.52	0.55 ± 0.07
T_V20h_	0.0631	0.20 ± 0.04	0.54	0.55 ± 0.07
T_V8hS_	0.0686	0.25 ± 0.05	–	0.52 ± 0.07
T_V12hS_	0.0934	0.29 ± 0.05	–	0.56 ± 0.07
T_V16hS_	0.0771	0.22 ± 0.03	–	0.49 ± 0.07
T_V20hS_	0.0778	0.22 ± 0.03	–	0.55 ± 0.07
RR	33.590	0.06 ± 0.01	0.19	0.61 ± 0.07
PS	0.0106	0.05 ± 0.01	0.16	0.47 ± 0.07
R_eff_	0.0001	0.03 ± 0.01	–	0.30 ± 0.10
BCS_Cal_	3.7127	0.29 ± 0.04	–	0.60 ± 0.07
BCS_Vis_	0.0710	0.25 ± 0.04	–	0.53 ± 0.07
HD	0.0577	0.25 ± 0.05	–	0.57 ± 0.09
BS	0.1156	0.33 ± 0.05	–	0.53 ± 0.06
EA	1151.7	0.40 ± 0.09	–	0.57 ± 0.12
EL	2.4413	0.32 ± 0.07	–	0.53 ± 0.12

*T*_*ES*_ ear skin temperature (°C), *T*_*SS*_ shoulder skin temperature (°C), *T*_*RS*_ rump skin temperature (°C), *T*_*TS*_ tail skin temperature (°C), *T*_*V4all*_ all measures (every 10 min) of vaginal temperatures for 4 days (°C), *T*_*V4days*_ four time measures of vaginal temperatures during 4 days (°C), *T*_*V8h*_ vaginal temperature measured at 8:00 h, during 4 days (°C); *T*_*V12h*_ vaginal temperature measured at 12:00 h, during 4 days (°C), *T*_*V16h*_ vaginal temperature measured at 16:00 h, during 4 days (°C), *T*_*V20h*_ vaginal temperature measured at 20:00 h, during 4 days, RR (°C): respiration rate/min; *PS* panting score; *R*_*eff*_ respiration efficiency; *BCS*_*Cal*_ caliper body condition score; *BCS*_*Vis*_ visual body condition score; *HD* hair density score; *BS* body size score; *EA* ear area (cm^2^); *EL* ear length (cm). *SD* standard deviation

### Genetic correlations

Estimates of genetic correlations among traits (${\mathrm{r}}_{\mathrm{g}}$) and their approximate standard error between pairs of traits are in Table 5. High positive ${\mathrm{r}}_{\mathrm{g}}$ were observed between SST traits, i.e. 0.78 (T_ES_ with T_SS_), 0.81 (T_SS_ with T_RS_, T_SS_ with T_TS_, and T_RS_ with T_TS_), and 0.91 (T_ES_ with T_RS_, and T_ES_ with T_TS_).

**Table 5:** Estimates of genetic correlations (upper diagonal) and their standard errors (lower diagonal) between vaginal temperatures measures in lactating sows under heat stress conditions

Traits	T_Vall_	T_V4days_	T_V8h_	T_V12h_	T_V16h_	T_V20h_	T_V8hS_	T_V12hS_	T_V16hS_	T_V20hS_
T_Vall_		0.99	0.91	0.96	0.96	0.77	0.95	0.95	0.94	0.89
T_V4days_	0.01		0.99	0.98	0.97	0.89	0.98	0.99	0.99	0.99
T_V8h_	0.04	0.01		0.99	0.96	0.96	1.00	0.88	0.75	0.77
T_V12h_	0.03	0.04	0.01		0.97	0.97	0.98	1.00	0.91	0.88
T_V16h_	0.03	0.03	0.03	0.01		0.99	0.90	0.95	0.99	0.97
T_V20h_	0.10	0.10	0.06	0.03	0.02		0.84	0.89	0.96	0.99
T_V8hS_	0.04	0.05	0.04	0.02	0.02	0.01		0.99	0.90	0.88
T_V12hS_	0.03	0.04	0.06	0.05	0.06	0.01	0.01		0.95	0.93
T_V16hS_	0.03	0.03	0.05	0.04	0.04	0.05	0.03	0.01		0.97
T_V20hS_	0.06	0.05	0.04	0.04	0.05	0.05	0.03	0.01	0.01	

*T*_*V4all*_ all measures (each 10 min) of vaginal temperatures for 4 days (°C), *T*_*V4days*_ four-time measures of vaginal temperatures during 4 days (°C), *T*_*V8h*_ vaginal temperature measured at 8:00 h, during 4 days (°C), *T*_*V12h*_ vaginal temperature measured at 12:00 h, during 4 days (°C), *T*_*V16h*_ vaginal temperature measured at 16:00 h, during 4 days (°C); *T*_*V20h*_ vaginal temperature measured at 20:00 h, during 4 days

Skin surface temperatures had low to moderate positive ${\mathrm{r}}_{\mathrm{g}}$ with T_V_ traits, ranging from 0.25 (T_TS_ with T_V12h_) to 0.88 (T_TS_ with T_V20hS_). Between all the T_V_ traits, T_Vall_ and single measurement records (i.e., T_V8hS_, T_V12hS_, T_V16hS_, and T_V20hS_) showed the highest ${\mathrm{r}}_{\mathrm{g}}$ estimates with SST traits (except T_Vall_ with T_TS_ with an ${\mathrm{r}}_{\mathrm{g}}$ of 0.30). Low ${\mathrm{r}}_{\mathrm{g}}$ estimates of 0.25, 0.24, 0.22, and 0.21 were observed for RR with T_ES_, T_SS_, T_RS_, and T_TS_, respectively. A similar pattern of low correlations was observed between SST and PS, with the ${\mathrm{r}}_{\mathrm{g}}$ between T_ES_ and PS being the highest (0.36), while the other trait combinations had estimates of 0.13, 0.16, and 0.17 (with T_SS_, T_RS_, and T_TS_, respectively). The SST traits were also lowly genetically correlated with BS, with estimates ranging from 0.05 (T_SS_ with BS) to 0.28 (T_ES_ with BS). Low ${\mathrm{r}}_{\mathrm{g}}$ were also estimated for SST with the other studied traits (see Additional file 1: Table S1).

Vaginal temperature traits were estimated to be positively and highly genetically correlated with each other. High ${\mathrm{r}}_{\mathrm{g}}$ (> 0.89) were estimated for T_Vall_ with the other T_V_ measures, except for T_Vall_ with T_V20h_ with an ${\mathrm{r}}_{\mathrm{g}}$ of 0.77. The same pattern was observed between T_V4days_ and T_V20h_ for which the ${\mathrm{r}}_{\mathrm{g}}$ was 0.89, while all the other combinations of T_V_ with T_V4days_ had an ${\mathrm{r}}_{\mathrm{g}}$ higher than 0.96 (see Table 5 for the trait combinations). Records at individual time points during the day (i.e., T_V8h_, T_V12h_, T_V16h_, and T_V20h_) were highly genetically correlated with each other, with values higher than 0.96. The same pattern of high ${\mathrm{r}}_{\mathrm{g}}$ was observed within the vaginal temperature traits based on single measurement records (i.e., T_V8hS_, T_V12hS_, T_V16hS_, and T_V20hS_), for which the later measurement times had the lowest ${\mathrm{r}}_{\mathrm{g}}$ (0.88 for T_V8hS_ with T_V20hS_), and the earlier measurement times had the highest ${\mathrm{r}}_{\mathrm{g}}$ (0.99 for T_V8hS_ with T_V12hS_, and 0.97 for T_V16hS_ with T_V20hS_). Vaginal temperature derived based on single records per day and single records had moderate to high ${\mathrm{r}}_{\mathrm{g}}$ with one another. Corresponding measurement times were highly genetically correlated with each other, with estimates equal to 1.0 for T_V8h_ with T_V8hS_, and T_V12h_ with T_V12hS_, and equal to 0.99 for T_V16h_ with T_V16hS_, and T_V20h_ with T_V20hS_. All other combinations of traits had a moderate to high ${\mathrm{r}}_{\mathrm{g}}$, ranging from 0.77 (T_V8h_ with T_V20hS_) to 0.98 (T_V12h_ with T_V8hS_).

The estimate of ${\mathrm{r}}_{\mathrm{g}}$ between RR and PS was 0.87 (± 0.02). For the other combinations of RR traits, the ${\mathrm{r}}_{\mathrm{g}}$ were low to moderate, with absolute values ranging from 0.16 (± 0.08) between RR and T_V8h_ to 0.42 (± 0.09) between RR and T_V16hS_, and between RR and T_V20Hs_ (± 0.07). Low ${\mathrm{r}}_{\mathrm{g}}$ were also estimated for PS, with absolute values ranging from − 0.01 (± 0.09) between PS and T_V4days_ to 0.87 (± 0.02) between PS and RR. Respiration efficiency (R_eff_) had positive moderate correlations of 0.41 (± 0.02), 0.36 (± 0.02), 0.56 (± 0.03), 0.56 (± 0.05), and 0.50 (± 0.05) with T_TS_, RR, PS, BCS_Cal_, and BCS_Vis_, respectively. Negative ${\mathrm{r}}_{\mathrm{g}}$ were found between R_eff_ and T_Vall_ (− 0.28 ± 0.11). R_eff_ was also negatively genetically correlated with T_V_ measured at 8:00 and 12:00 h, with values of − 0.38 (± 0.07), − 0.20 (± 0.07), − 0.35 (± 0.10), and − 0.17 (± 0.10) for T_V8h_, T_V12h_, T_V8hS_, and T_V12hS_. However, R_eff_ had positive correlations with T_V_ measured at 16:00 and 20:00 h, with values of 0.19 (± 0.08), 0.19 (± 0.11), 0.05 (± 0.08), and 0.07 (± 0.09) for T_V16h_, T_V20h_, T_V16hS_, and T_V20hS_.

A high ${\mathrm{r}}_{\mathrm{g}}$ of 0.92 (± 0.04) was estimated between BCS_Cal_ and BCS_Vis_. Negative low to moderate ${\mathrm{r}}_{\mathrm{g}}$ were estimated between BCS_Cal_ and T_V_ measurements, with values ranging from − 0.32 (± 0.08) for BCS_Cal_ with T_V12hS_ to − 0.55 (± 0.10) for BCS_Cal_ with T_V8h_. A low negative ${\mathrm{r}}_{\mathrm{g}}$ was estimated between BCS_Cal_ and SST measures, ranging from -0.08 (± 0.03) for BCS_Cal_ with T_TS_ to -0.19 (± 0.04) for BCS_Cal_ with T_RS_. Similar to BCS_Cal_, BCS_Vis_ had low negative ${\mathrm{r}}_{\mathrm{g}}$ with SST (from − 0.02 ± 0.04 with T_TS_ to − 0.12 ± 0.04 with T_RS_). Negative low to moderate ${\mathrm{r}}_{\mathrm{g}}$ were estimated between BCS_Vis_ and T_V_ traits (from − 0.27 ± 0.08 with T_V20hS_ to 0.49 ± 0.08 with T_Vall_). Body size was moderately genetically correlated with BCS_Cal_ and BCS_Vis_, with estimated values of 0.60 (± 0.05) and 0.63 (± 0.06), respectively. The ${\mathrm{r}}_{\mathrm{g}}$ between BS and SST traits ranged from 0.05 (± 0.08) for BS with T_RS_ to 0.28 (± 0.09) for BS with T_ES_. Low ${\mathrm{r}}_{\mathrm{g}}$ were also estimated between BS and T_V_ traits, with absolute values ranging from − 0.03 ± 0.08 (with T_V12h_) to 0.21 ± 0.08 (with T_V20hS_). For HD, the ${\mathrm{r}}_{\mathrm{g}}$ were negative and low with T_ES_ (− 0.02 ± 0.03), T_SS_ (− 0.06 ± 0.04), and T_RS_ (-0.02 ± 0.04), and positive with T_TS_ (0.26 ± 0.04). Low ${\mathrm{r}}_{\mathrm{g}}$ were also estimated between HD and T_V_ traits, ranging from 0.08 (for HD with T_V12hS_ [± 0.08] and T_V16hS_ [± 0.07]) to 0.24 ± 0.11 (for HD with T_Vall_). All other combinations of traits presented low ${\mathrm{r}}_{\mathrm{g}}$ and are in Additional file 1: Table S1.

### Theoretical accuracy

The average theoretical accuracy of GEBV was moderately high for all the studied traits (Table 4); the GEBV for R_eff_ had the lowest accuracy (0.30 ± 0.10), and T_Vall_ the highest (0.66 ± 0.07). The GEBV for SST traits had similar accuracies, ranging from 0.58 ± 0.07 for T_SS_ to 0.60 ± 0.07 for T_ES_. Among all the T_V_-based traits, T_Vall_ had the highest accuracy (0.66 ± 0.07).

## Discussion

### Heritability and repeatability estimates

Advances in genetic selection in the pig industry have led to increased productivity, which has resulted in sows producing more heat that they must dissipate without too much effort to avoid heat stress. To cope with elevated body temperatures, lactating sows tend to reduce feed intake and milk production, which leads to compromised reproductive performance and growth rate of the piglets [2, 41, 42]. One of the starting points of genetic studies that aim at selecting more heat-tolerant animals is to define heritable phenotypes that are associated with resistance or susceptibility to heat stress [43]. In pigs, genetic parameters for physiological heat stress indicators have rarely been estimated, especially in lactating sows. To the best of our knowledge, the present study is the first to evaluate physiological indicators of heat stress in a large commercial pig population.

The genetic variation observed for the traits evaluated in this study indicates great opportunities for genetic improvement of heat tolerance in swine populations. T_V_, which represents the core body temperature, displayed a wide range of heritability estimates (from 0.15 to 0.29) depending on the time of measurement. Gourdine et al. [44] estimated a heritability of 0.35 ± 0.09 for rectal temperature measured twice a day from farrowing to day 32 of lactation in purebred Large White sows. Other studies estimated the heritability of core body temperature using rectal measures at different life stages, ranging from 0.02 to 0.58 in newborn piglets (Iberian x Meishan crossbred; [45]), and from 0.07 to 0.34 in growing pigs at 23 weeks of age (Large White x Creole; [46]). Regarding the measures of T_V_ evaluated in our study, T_Vall_, which accounts for all T_V_ measures, had the lowest heritabilities. This lower heritability of T_Vall_ might be related with the type of model used (i.e., a repeatability model), which may not be able to account for reduced genetic variation in T_V_ measured during the night period. One alternative way to analyze this longitudinal data may be to use random regression models, where the amount of genetic variation could be estimated for each time-point. The single measure traits, T_V8hS_ and T_V12hS_, had the highest estimates of heritability (0.25 and 0.29, respectively) among all the T_V_ and could be used in breeding programs to select individuals that can maintain their body temperature in different weather conditions due to its ease and speed of collection, and the large amount of genetic variation that they account for. Furthermore, the high genetic correlations and Spearman correlations greater than 0.82 between GEBV of the different T_V_ measurements indicate that all T_V_ metrics were able to similarly rank the individuals based on their GEBV, and that in most cases the same animal would be selected based on their high or low GEBV.

Increasing RR and beginning to pant is one strategy that pigs use to release more heat through evaporative heat loss [47]. However, these two traits were lowly heritable (0.06 and 0.05) in the studied population, but with a heritability significantly different from 0. Kim [18], estimated a higher heritability (0.39 ± 0.13) for RR in pre-puberal gilts from a cross between a PIC maternal and a Duroc line than those found in the current study. This difference in heritability estimates between these two studies might be because the individuals from the two studied populations were evaluated at different life stages, because of differences in how traits were measured, or because heritability is a population-specific parameter that can vary depending on the allele frequencies in each population. In addition, when analyzing RR in Holstein cows, Luo et al. [26] also found a low heritability of 0.04 ± 0.01.

Respiration efficiency, which was defined in our study as the efficiency of the animal to reduce its body temperature through increased respiration rate, also provided low heritability estimates, but significantly different from 0. Other factors that were not considered in our study can affect the animal's metabolism, such as reduced feed consumption, reduced milk production, increased water usage, and changes in the prioritization of body reserves, and consequently the animal’s ability to cope with changes in body temperature and influence R_eff_. As a result, the estimation of R_eff_ may be inaccurate and future studies should consider effects such as feed consumption and milk production in their calculations.

The lack of functional sweat glands in pigs results in most of the heat elimination by evaporation to occur in the respiratory tract. Still, the skin represents another mechanism that pigs use for heat loss by increasing blood flow to the skin. However, this mechanism is inefficient during heat stress events, when the ambient temperature might approach or exceed the skin temperature and, thus, instead of losing heat to the environment the skin ends up gaining heat [48]. The low heritability estimated for SST traits provides evidence for the large environmental impact on these traits. In addition, SST traits presented a similar amount of additive genetic variance but had estimates of r_e_ that differed depending on the combination of traits, and for T_ES_ it implies that the phenotypic values changed substantially within the day and that it is more responsive to changes in the thermal environment. In addition, SST traits may be substantially affected by cooling systems (evaporative or conductive), water (e.g., sows play with water to wet their skin and increase heat loss by evaporation), and air speed [49]. Therefore, SST may have little utility in determining the severity of heat stress due to this large environmental effect, compared to T_V_.

In this study, body size score was demonstrated to be highly heritable (mean heritability of 0.33), which is consistent with other studies that evaluated similar traits in swine populations. Ohnishi and Sato [50] in the Duroc breed and Johnson and Nugent [51] in the Landrace breed found a similar heritability of 0.32 for body length. The difference between those two studies and our study is that they used a tape measure to measure body length, while we scored BS visually, which is faster and easier, yet resulted in a similar estimate of heritability.

Genetic parameters for ear size are scarce in the literature. In the current study, ear size measurements were found to be a highly heritable trait. Potentially, larger ears could facilitate heat loss to the environment since the animal’s skin surface area would be greater compared with a same sized individual with smaller ears [7]. In animals with an increased surface area to body mass ratio, heat loss is, in general, greater than heat gain at higher temperatures. Hair density is another trait that can influence the ability of the animal to dissipate heat through the skin which, to our knowledge, has not been previously reported in the literature. Therefore, we decided to evaluate this easy-to-measure trait based on comparative biology and previous literature in cattle with a slick coat, as well as the scientifically-established notion that increased hair cover may reduce the ability of mammals to dissipate excess body heat and contribute to heat stress sensitivity. While hair length can be a contributor to hair density, they may not be directly associated because hair density is primarily influenced by the total number of hair follicles. When considering the effects of convective heat loss (air flow over the skin), one would expect that a pig with long but sparse hair would be better able to dissipate body heat since total hair cover would not greatly impair the heat loss mechanisms, whereas a pig with shorter but denser hair would have reduced air flow through the skin. Based on the moderate estimate of heritability observed for hair density score, future studies should evaluate both hair density and hair length, using more objective measurements. In cattle, a slick coat is associated with higher thermotolerance under a tropical environment [52–54]. For example, Senepol cattle with slick hair can maintain a lower body temperature compared to individuals of the same breed without slick hair. Thus, it is possible that sows with more hair cover may have a disadvantage when dissipating heat through the skin. Due to the ease of its measurement and its moderate heritability estimates, genetic progress for HD could be achieved in this swine population, but the impact on heat tolerance would be limited.

BCS measures presented moderate to high heritabilities. Body condition is an essential measure to ensure the right amount of nutrition and consequently production in the herd [55]. Fat and thin sows can reduce the profitability of the herd, since they can have reduced farrowing rates, longer time between weaning and estrus, and fewer piglets per litter, which affects sow productivity, and consequently, herd profitability [56, 57]. Maintaining optimal BCS through lactation can help sows perform to their full potential in extreme heat, provide enough nutrients to their offspring, and prepare the sow for the next gestation [58]. Both visual and caliper BCS measures present advantages and disadvantages. Measuring a phenotype visually needs practice and can vary from scorer to scorer when not properly trained. Furthermore, an ideal BCS for one farm may not be considered as ideal for another farm. On the one hand, visually-scored BCS is a simple, and low-cost measure that may be easier to implement on-farm. On the other hand, using a caliper represents a standardization measure for the animal's body score and will always give the same value, regardless of the farm. However, using this tool also requires proper training, since it must be positioned in the correct place on the animal's body, which makes it difficult when the animal is stressed and moves. Caliper and visually-scored BCS also presented moderate to high correlations up to 0.71, meaning that the BCS values agree well between the two metrics. The moderate to high heritabilities estimated in this study suggest that both BCS measurements can be used to genetically select individuals with similar accuracy.

### Genetic correlations

Considering the estimates of genetic correlations between measurements of T_V_, it can be concluded that selection on any of the measurement (trait) chosen will result in substantial genetic progress for all the T_V_ traits. Among all the combinations of measurements, those taken at more distant times (e.g., T_V8hS_ and T_V20hS_) had the lowest genetic correlation (although > 0.75) compared to measurements taken at closer times (e.g., T_V8hS_ and T_V12hS_). This was expected, as behavioral, physiological, and metabolic changes tend to be observed at different hours of the day, and may require activation of different sets of genes. Events such as feeding increase the metabolic heat produced by an animal's body due to the heat associated with nutrient processing [59, 60] and, consequently, the need to activate mechanisms to remove this metabolic heat. In the afternoon, the effect of the accumulated heat (metabolic and environmental) may influence the animal by activating different sets of genes. With regard to selection, the rank of individuals based on their GEBV was similar for all T_V_ traits. Thus, they represent potentially useful traits to be included in a selection scheme for heat tolerance, especially T_V_ measured at noon (both repeated and single day record) due its higher heritability estimate.

Skin surface temperatures measured at different locations on the sow’s body were highly genetically correlated (from 0.78 to 0.91). This was expected due to the role of the skin’s surface in dissipating heat [48, 49]. In addition to the genetic mechanism underlying each trait, the environment might influence these correlations. For example, animals that are wetted by sprinklers and combined with elevated airspeeds are more efficient at losing heat by evaporation [49], but this is more related to management practices than to the animal's genetics. The low heritability obtained for skin surface temperature traits show that they are largely influenced by environmental variations that might not have been properly accounted for by the systematic effects included in the models (e.g., variability in the effectiveness of heat mitigation strategies, location of the pen within a barn, differential air speed). Estimates of the genetic correlations between SST and body temperature varied greatly depending on the T_V_-based trait used. In most cases, genetic correlation estimates were low, except when using single T_V_ measurements. The low genetic correlation obtained with repeated measurements shows that SST may not be the best indicator trait for heat tolerance, since, as mentioned earlier, SST can be more affected by several external factors.

The ${\mathrm{r}}_{\mathrm{g}}$ of SST with R_eff_ was low, which indicates a weak genetic association of most SST traits with the animals’ efficiency for losing heat. SST also had weak genetic correlation estimates with RR and PS. However, T_TS_ had a higher ${\mathrm{r}}_{\mathrm{g}}$ with R_eff_. This genetic relationship might be related to genes that act on both phenotypes that could be further investigated based on genome-wide association studies and multi-omics studies (e.g., transcriptomics, metabolomics). However, as Gourdine et al. [43] pointed out, studies that identify quantitative trait loci for thermoregulation traits in pigs are scarce, and much effort is needed to increase the number of pigs for which both genomic and phenotypic information for relevant traits is available.

Genetic correlations between T_V_ and RR and between T_V_ and PS had a similar pattern, with the single T_V_ records presenting a higher ${\mathrm{r}}_{\mathrm{g}}$ compared to the repeated records. The ${\mathrm{r}}_{\mathrm{g}}$ between respiration rate and PS was low when compared to the other measured traits, such as BCS (visual and caliper), HD, ear measures and BS. RR and PS were highly correlated with each other. Given its ease of measuring, PS might represent a potential trait to be used for selecting heat-tolerant individuals. However, since the role of panting is most significant when an animal is severely heat-stressed, if PS was included in a breeding scheme, it should be recorded during the hottest period of the day to allow for better recording of the score categories.

Ear size (area and length) was lowly genetically correlated with the majority of the traits evaluated. However, EL had a moderate negative correlation with R_eff_, meaning that longer ears are genetically related to a better R_eff_. This relationship might not be directly related to the SST measured at the ear, since the ${\mathrm{r}}_{\mathrm{g}}$ between EL and T_ES_ was low (0.08), however, it may be related to sensitization mechanisms, where individuals with larger ears can better perceive fluctuations in their body temperature and trigger responses on the hypothalamus and a consequent increase in their respiration. This may be related to the fact that we estimated a positive genetic correlation between BS and EL. Therefore, greater BS is genetically related to greater EL, and these individuals tend to have a higher RR (${\mathrm{r}}_{\mathrm{g}}$ between BS and RR is equal to 0.21) and better R_eff_ (${\mathrm{r}}_{\mathrm{g}}$ between BS and R_eff_ is equal to − 0.29). However, future studies need to further investigate this relationship. Genetic correlations between BS and BCS and between BS and T_V_, agree with this finding, showing that greater BS is genetically related to greater BCS, which is genetically related to lower T_V_. Another explanation for the observed genetic correlations between BCS and R_eff_ could be that, as respiratory responses are controlled by thermoreceptors located in the skin and muscles, fatter sows may feel the effects of increased temperature faster, which triggers the reaction of the receptors that increase RR. This would result in a lower core body temperature and better R_eff_. However, this relationship should be further investigated.

The positive (but low) ${\mathrm{r}}_{\mathrm{g}}$ estimated between HD and T_V_ measures might indicate that individuals with more hair cover have a disadvantage for dissipating heat and consequently increase their body temperature under heat stress conditions. As stated previously, the unfavorable genetic relationship between hair cover and thermotolerance was proven true in Senepol cattle [52–54]. Further studies are needed to investigate this genetic relationship between hair cover and T_V_ and understand whether sows with more hair cover have a disadvantage (or not) when dissipating heat through the skin. If this relationship was proven, HD could be used as another indicator for thermotolerance in breeding schemes.

### Potential implications and limitations

This is the first study to genetically investigate phenotypes that are related to physiological responses of sows under heat stress conditions in commercial settings. We report estimates of genetic parameters for traits related to physiological responses that provide important findings to the literature. The heritability and substantial additive genetic variance of the studied traits show the potential of including them in selection schemes to improve heat tolerance in swine. However, economically-important phenotypes that reflect the productive and reproductive performance of sows (e.g., litter weight at weaning, subsequent litter size and weight, interval between weaning to subsequent estrus, and feed efficiency) need to be collected and the genetic correlations of the studied traits with these economically-important traits quantified, enabling the development of a selection sub-index based on a combination of some of the traits evaluated in this study, in order to improve heat tolerance while maintaining productivity. In addition, a larger number of phenotyped and genotyped animals in other populations is needed to validate the results obtained. This validation is also important in purebred populations, which are usually the populations under intense selection pressure. Furthermore, additional studies should be performed to evaluate the genetic mechanisms underlying the main traits evaluated in this study (i.e., vaginal temperature, respiration rate, panting score, and respiration efficiency). Finally, different statistical methods, such as random regression models, should be tested to better account for the genetic and environmental variations throughout the collection period.

## Conclusions

The estimates of heritability obtained here demonstrate that genetic progress for thermotolerance can be achieved by including the studied traits in selection schemes. Low (but significant) heritabilities were estimated for SST traits, RR, PS, and R_eff_. Vaginal temperature presented moderate heritabilities, with T_V_ measured at 8:00 and 12:00 h being the most heritable, while T_Vall_ had the lowest heritability among the T_V_ traits evaluated. Moderate to high heritabilities were observed for BCS measures, HD, BS, EA, and EL, indicating great potential for genetic progress on these traits. Vaginal temperature and R_eff_ are directly related with the animal’s response to thermal adaptation and are key traits to be used in the selection of more heat-tolerant animals. However, such selection for these traits must be balanced against the ease of measurement, genetic correlations with other economically-important traits (e.g., performance, health, longevity), and their contribution to the breeding objectives. Furthermore, the results for the genetic correlations provide evidence of the complex relationships among these traits and confirm the importance of developing a thermotolerance selection sub-index to breed for improved heat tolerance in pigs. Finally, additional investigation is needed, including the evaluation of other statistical models (e.g., random regression model) and studies to unravel the genetic background of thermotolerance traits.

### Supplementary Information

**Additional file 1: Table S1.** Genetic correlations (upper diagonal) and standard deviation (lower diagonal) estimations among physiological indicators of heat stress in lactating sows under heat stress conditions.

## Data Availability

All the data supporting the results of this study are included in the article and the Additional files. The raw phenotypic and genotypic data cannot be shared because they are owned by commercial breeding companies and this information is commercially sensitive.
